# Acute Promyelocytic Leukemia With PML‐RARA Rearrangement: Clinical and Morphologic Features

**DOI:** 10.1002/ccr3.73830

**Published:** 2026-10-30

**Authors:** Chukwuka Elendu, Ayodeji D. Johnson, Ayi T. Debua

**Affiliations:** ^1^ Federal University Teaching Hospital Owerri Nigeria; ^2^ Boston Medical Center Brighton Michigan USA; ^3^ University of Calabar Teaching Hospital Calabar Nigeria

**Keywords:** acute promyelocytic leukemia, all‐trans retinoic acid, disseminated intravascular coagulation, hypergranular promyelocytes, PML‐RARA rearrangement

## Abstract

Recognition of the characteristic morphologic features of acute promyelocytic leukemia on peripheral blood smear should prompt immediate initiation of all‐trans retinoic acid while awaiting confirmatory testing, as early intervention is critical to reducing hemorrhagic mortality and improving clinical outcomes.

## 1 Case Description

A 10‐year‐old boy presented with a 2‐week history of progressive fatigue, low‐grade fever, intermittent gum bleeding, easy bruising, and reduced activity. On examination, the child appeared pale with scattered ecchymoses but no significant lymphadenopathy or hepatosplenomegaly. Laboratory evaluation revealed pancytopenia (hemoglobin 7.8 g/dL, leukocyte count 2.1 × 10^9^/L, platelet count 32 × 10^9^/L). Peripheral blood smear demonstrated abnormal promyelocytes with hypergranular cytoplasm and occasional Auer rods (Figure 1), suggestive of the hypergranular variant of acute promyelocytic leukemia (APL). Coagulation studies showed a prolonged prothrombin time (PT 19.8 s; INR 1.8), activated partial thromboplastin time (aPTT 46.2 s), elevated D‐dimer (> 20.0 μg/mL FEU), and hypofibrinogenemia (fibrinogen 0.9 g/L), consistent with disseminated intravascular coagulation.

**FIGURE 1:**
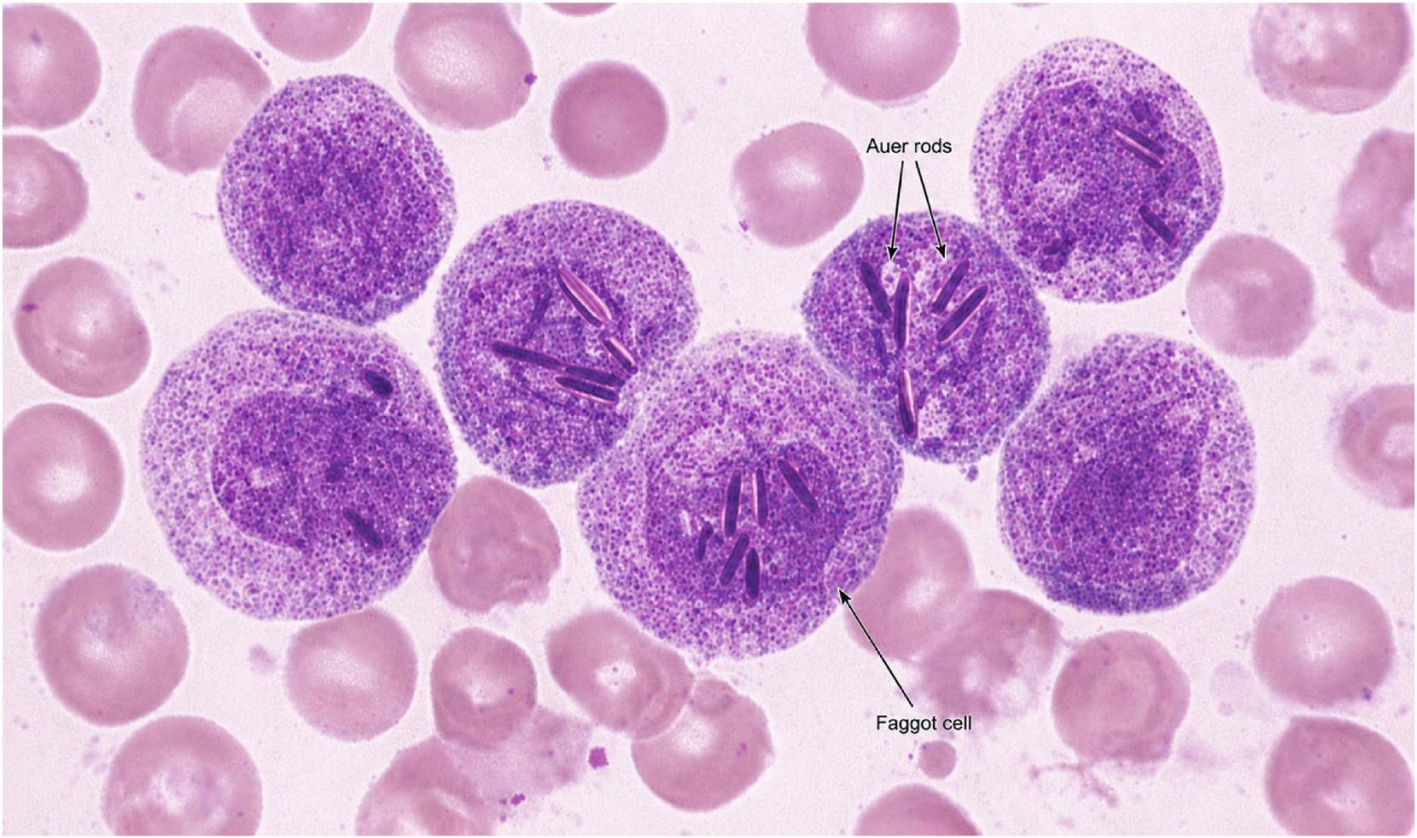
Peripheral blood smear (Wright–Giemsa stain, ×1000 magnification) demonstrating hypergranular promyelocytes. Arrows labeled “Auer rods” indicate characteristic needle‐shaped azurophilic cytoplasmic inclusions. The arrow labeled “Faggot cell” identifies a hypergranular promyelocyte containing numerous bundled Auer rods, a classic morphologic feature of acute promyelocytic leukemia.

In children presenting with cytopenias and bleeding diathesis, differential diagnoses include acute lymphoblastic leukemia, other acute myeloid leukemia subtypes, aplastic anemia, and severe infection with disseminated intravascular coagulation. However, the combination of these morphologic features and coagulopathy strongly suggested APL. Bone marrow examination confirmed a predominance of abnormal promyelocytes (Figure 2), and flow cytometry demonstrated an immunophenotype consistent with APL, characterized by expression of CD13, CD33, and myeloperoxidase (MPO), with absent CD34 and HLA‐DR expression. Conventional cytogenetic analysis identified the t(15;17)(q24;q21) translocation, while fluorescence in situ hybridization (FISH) confirmed the PML‐RARA rearrangement and reverse transcription polymerase chain reaction (RT‐PCR) detected the PML‐RARA fusion transcript, establishing the diagnosis of APL.

**FIGURE 2:**
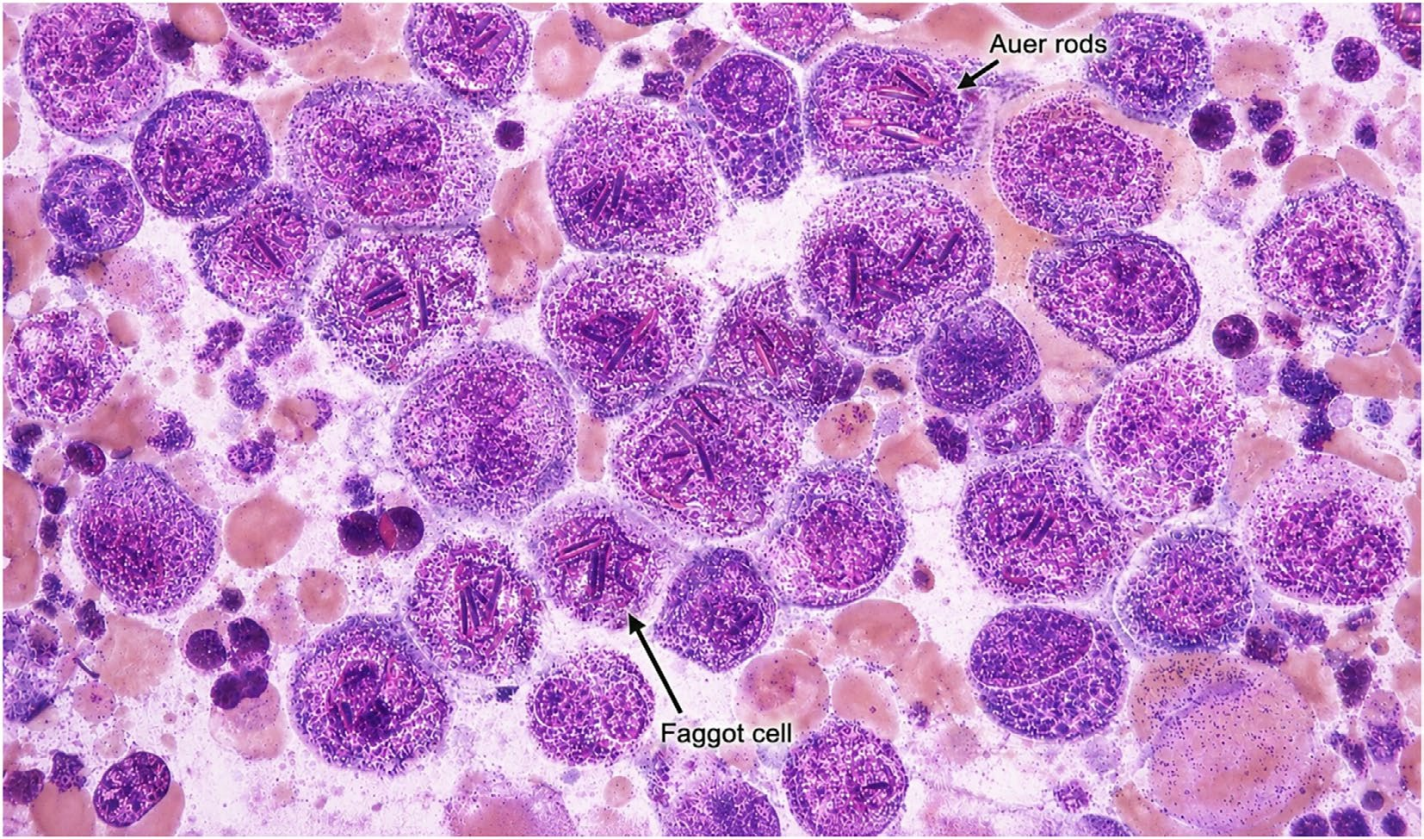
Bone marrow aspirate (Wright–Giemsa stain, ×1000 magnification) demonstrating numerous abnormal hypergranular promyelocytes. The arrow labeled “Auer rods” indicates characteristic needle‐shaped azurophilic cytoplasmic inclusions, while the arrow labeled “Faggot cell” identifies a hypergranular promyelocyte containing numerous bundled Auer rods, a classic morphologic feature of acute promyelocytic leukemia.

All‐trans retinoic acid (ATRA) was initiated upon clinical suspicion, prior to cytogenetic confirmation, because of the high risk of early hemorrhagic complications, together with aggressive supportive transfusion therapy to maintain platelet counts and fibrinogen levels. Following diagnostic confirmation, the patient received induction therapy with ATRA plus arsenic trioxide without additional chemotherapy, consistent with the management of low‐risk APL. The patient demonstrated rapid clinical and hematologic improvement with resolution of bleeding manifestations and normalization of coagulation parameters. Complete molecular remission, confirmed by negative RT‐PCR for the PML‐RARA fusion transcript, was documented after completion of induction therapy, with no evidence of differentiation syndrome during treatment.

## 2 Discussion

APL is a hematologic emergency in which recognition of hypergranular promyelocytes with multiple Auer rods (“faggot cells”) should immediately raise suspicion for the diagnosis [1]. Because early mortality is primarily attributable to hemorrhage secondary to consumptive coagulopathy, ATRA should be initiated as soon as APL is clinically suspected, without awaiting confirmatory testing [1]. Definitive diagnosis is established by demonstrating the PML‐RARA rearrangement using cytogenetic and molecular techniques [2]. Recognition of these characteristic morphologic features remains essential for early therapeutic decision‐making and has contributed to the marked improvement in clinical outcomes observed in APL [1, 3]. The present case highlights the diagnostic value of recognizing characteristic promyelocyte morphology in a child presenting with severe coagulopathy. The coexistence of hypergranular promyelocytes, Auer rods, and disseminated intravascular coagulation provided a strong clinical and morphologic clue to APL, while molecular confirmation established the diagnosis. The rapid hematologic and coagulation response following ATRA and arsenic trioxide further illustrates the clinical benefit of early diagnosis and targeted differentiation therapy in children, helping to prevent fatal hemorrhagic complications.

## Author Contributions

**Chukwuka Elendu:** conceptualization, investigation, data curation, methodology, formal analysis, writing – original draft, writing – review and editing, validation, visualization, supervision, project administration. **Ayodeji D. Johnson:** validation, visualization. **Ayi T. Debua:** writing – review and editing.

## Funding

The authors have nothing to report.

## Ethics Statement

Ethical approval was not required in accordance with institutional policy.

## Consent

Written informed consent was obtained from the patient's parent/legal guardian, and assent was obtained from the patient for publication of this report and accompanying clinical image.

## Conflicts of Interest

The authors declare no conflicts of interest.

## Data Availability

Data sharing is not applicable to this study because no new data were generated.
